# Social interactions affecting caste development through physiological actions in termites

**DOI:** 10.3389/fphys.2014.00127

**Published:** 2014-04-09

**Authors:** Dai Watanabe, Hiroki Gotoh, Toru Miura, Kiyoto Maekawa

**Affiliations:** ^1^Laboratory of Ecological Genetics, Graduate School of Environmental Science, Hokkaido UniversitySapporo, Hokkaido, Japan; ^2^Department of Biology, Graduate School of Science and Engineering, University of ToyamaToyama, Japan; ^3^Department of Entomology, Washington State UniversityPullman, WA, USA

**Keywords:** termite, social physiology, social interaction, juvenile hormone, caste differentiation, soldier differentiation

## Abstract

A colony of social insects is not only an aggregation of individuals but also a functional unit. To achieve adaptive social behavior in fluctuating environmental conditions, in addition to coordination of physiological status in each individual, the whole colony is coordinated by interactions among colony members. The study on the regulation of social-insect colonies is termed “social physiology.” Termites, a major group of social insects, exhibit many interesting phenomena related to social physiology, such as mechanisms of caste regulation in a colony. In their colonies, there are different types of individuals, i.e., castes, which show distinctive phenotypes specialized in specific colony tasks. Termite castes comprise reproductives, soldiers and workers, and the caste composition can be altered depending on circumstances. For the regulation of caste compositions, interactions among individuals, i.e., social interactions, are thought to be important. In this article, we review previous studies on the adaptive meanings and those on the proximate mechanisms of the caste regulation in termites, and try to understand those comprehensively in terms of social physiology. Firstly, we summarize classical studies on the social interactions. Secondly, previous studies on the pheromone substances that mediate the caste regulatory mechanisms are overviewed. Then, we discuss the roles of a physiological factor, juvenile hormone (JH) in the regulation of caste differentiation. Finally, we introduce the achievements of molecular studies on the animal sociality (i.e., sociogenomics) in terms of social physiology. By comparing the proximate mechanisms of social physiology in termites with those in hymenopterans, we try to get insights into the general principles of social physiology in social animals.

## An example of social physiology: caste regulatory mechanisms in termites

“Physiology” refers to biological studies that deal with the functions and activities of living things. Most of physiological studies focus on the mechanisms in an individual or part(s) of an individual (e.g., Randall et al., 2002). However, physiological events are not necessarily limited to within an individual, but may also involve interactions with environments surrounding the individual. In some organisms, by interacting and cooperating with other individuals of the same species, a sophisticated entity is constructed at a higher dimension than the individual level. Such an entity is often referred to as a “society” (Wilson, 1975), and a number of studies on social organisms, particularly social insects, have been performed. During the last two decades, the term “social physiology” was proposed to describe the study that tried to understand communication systems that facilitate colony activities (Seeley, 1995).

A colony of social insects is often referred to as a “superorganism” as if it were one individual (Hölldobler and Wilson, 2008). As this name suggests, colonies can change their inner conditions through various actions performed by colony members. These performances by colony members can be divided into two types of actions: the actions of each individual (division of labor) and the interactions between colony members (communications). Two study areas, i.e., social anatomy and social physiology, that were firstly classified by Johnson and Linksvayer (2010) respectively focus on the proximate factors responsible for the two types of actions. They also argued that these two types of studies should be deeply connected to each other although studies on social physiology were delayed compared to social anatomy. Furthermore, studies in social physiology have been concentrated especially in social hymenopterans, i.e., ants, bees and social wasps (Seeley, 1995; Pankiw, 2004; Johnson and Linksvayer, 2010). However, this study area should include examples in broad range of animal groups in order to elucidate the principles of social physiology. In that sense, another major eusocial insect group, termites, should also be focused as the study materials of social physiology.

Castes, one of the distinctive features of social insects, possess morphologies specialized in tasks which are allocated in a colony (Wilson, 1971). The regulatory mechanisms optimizing caste ratios, including behavioral castes, is an essential focus in social physiology because it maximizes the colony productivity (Johnson and Linksvayer, 2010). To discuss caste regulatory mechanisms, termites are an interesting group due to a number of their conspicuous characteristics (Howard and Thorne, 2011). For example, termites construct diverse, huge, and complex nests, which include enormous numbers of individuals (Wilson, 1971; Noirot and Darlington, 2000). In termites, therefore, there must be distinctive mechanisms to coordinate such huge colonies, in comparison with those of social hymenopterans. Furthermore, termites possess the soldier caste, that is distinctive among social insects due to their developmental status and specialized morphologies (Deligne et al., 1981; Roisin, 2000). The soldier caste is thought to be an immature (non-imago) stage, probably due to the hemimetabolous caste developmental pathways (Eggleton, 2011) in termites, while social hymenopterans are holometabolous (Miura, 2004). Thus, although social hymenopterans have provided us implications, distinctive characteristics in termites will also provide new insights into the principles of social physiology.

In this article, we aim to overview the knowledge on the proximate mechanisms of caste regulations in termites as an example of social physiology, especially focusing on the soldier differentiation. We would like to discuss this issue along the biological events that occur in termite colonies and inside the focal individuals: from the communications among colony members, via physiological actions, affecting the developmental events. Furthermore, the molecules responsible for these processes are also focused.

## Caste systems in termites

### Factors affecting the caste developmental fates

**Figure 1:**
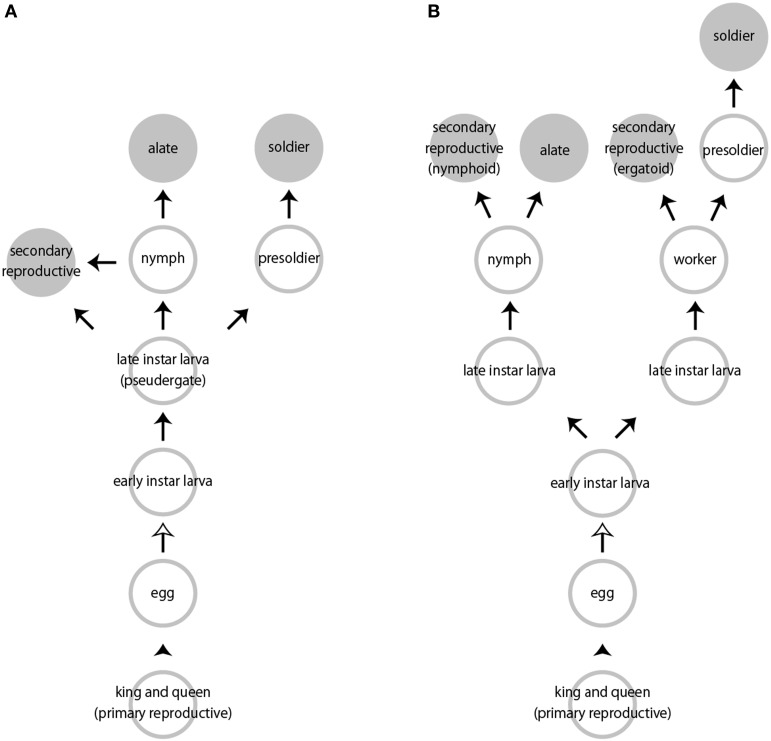
**Representative caste developmental patterns in termites. (A)** Linear pathway. **(B)** Bifurcated pathway. Arrowheads indicate mating and oviposition. White and black arrows indicate hatching and molts, respectively. Not all pseudergates or workers molt into subsequent developmental stages.

Caste differentiation in social insects is a representative case of polyphenism, in which multiple discrete phenotypes are seen in a single species (Nijhout, 1999, 2003). Castes of termites are primarily divided into two types of individuals: fertile (reproductives) and sterile individuals (neuters) (Thorne, 1996; Roisin, 2000). The sterile castes include workers, presoldiers and soldiers, while the fertile ones are alates, primary reproductives and secondary reproductives (Figure 1). Alates with imaginal characteristics (i.e., wings, testes, ovaries) that develop during the nymphal stages perform nuptial flights, shed their wings, mate, found new colonies, and then become primary reproductives (i.e., kings and queens). When primary reproductives die or become scenescent, or when colonies expand, some individuals differentiate into the secondary reproductives.

In contrast, soldiers and workers are sterile castes not engaged in reproduction. Late-stage larvae that behave as workers are called as “pseudergates” in the relatively basal termite lineages (Thorne, 1996). In this review, we will expediently use the term “worker” including pseudergate *sensu lato* (Korb and Hartfelder, 2008; Roisin and Korb, 2011). A part of workers differentiate into soldiers with drastic morphological modifications. A soldier possesses a pair of extremely elongated mandibles and/or a frontal projection with an exocrine gland from which defensive substances are discharged (Noirot, 1969; Weesner, 1969).

Depending on the species, the developmental pathways are roughly divided into two patterns, i.e., a linear pathway or a bifurcated pathway (Roisin, 2000; Bourguignon et al., 2009; Figure 1). Basically, all larvae hatched from eggs have the potential to develop into any castes. Classically, assuming that all egg contains the similar or identical genetic information, caste developmental fates are determined depending on various environmental factors experienced during postembryonic development, as well as sex and egg factors (Noirot, 1990, 1991). Major environmental stimuli include nutritional and seasonal factors such as temperature and humidity (Noirot, 1991; Fei and Henderson, 2002; Liu et al., 2005; Scharf et al., 2007). Termites can receive these stimuli by themselves, and in some cases, this environmental information can be transferred through social interactions such as allogrooming and/or trophallaxis, the latter of which is the exchange of nutritious food by stomodeal (i.e., oral) or proctodeal (i.e., anal) transfer (Noirot, 1991; Tarver et al., 2011). However, little is known about the relationships between environmental factors and patterns of social interactions (e.g., Machida et al., 2001).

Except for environmental stimuli, sexes are also important factors for the caste differentiation (Noirot, 1990). Termites show the XY sex determination system (Matsuura, 2011), and neuter castes are specialized in or biased toward either sex in some species (Roisin, 2000). For instance, in the genus *Nasutitermes* only male workers differentiated into soldiers (Hojo et al., 2004). In addition, a candidate X-chromosome-linked gene, *worker*, is reported to affect the caste differentiation in *Reticulitermes speratus* (Hayashi et al., 2007). The *worker* is hypothesized to have two alleles (*wk^A^* and *wk^B^*). They reported that male (XY) larvae possessing *wk^A^* were destined to be workers, while in female (XX), heterozygous in *worker* locus determined the differentiation into workers. Larvae with other genotypes basically develop into nymphs although only the genotype for females with homozygous *wk^B^* is thought to be lethal. This is the first report showing a genetic influence in reproductive division of labor (Kitade et al., 2011). However, it should also be noted that the caste fates are also affected by coexisting castes (Hayashi et al., 2007), suggesting that, except for environmental cues, social interaction among castes (or phenotypes) is the major factor affecting the caste composition in termites. The abnormal caste ratios set by artificial manipulations were gradually returned to the normal caste ratios just by keeping those colonies under laboratory conditions (Lefeuve and Bordereau, 1984; Mao et al., 2005; Park and Raina, 2005). These experiments were performed under laboratory conditions where environmental conditions were stable, so that the changes of social interactions are suggested to be the important factors adjusting the caste ratio.

### Social interactions controlling caste ratio

Historically, studies on the caste ratio regulations in termites started with the reports on seasonal transitions of caste composition in some species, such as *Odontotermes redemanni* (Banerjee, 1966) and *Trinervitermes ebenerianus* (Sands, 1965). Howard and Haverty (1981) found that, in *Reticulitermes flavipes*, the caste proportion in a colony changed with seasons. They reported that the soldier ratios increased concurrently with temperature and peaked when alate differentiation was about to occur. Furthermore, the ratios of soldiers and reproductives were kept at a low level (2 and 0.1%, respectively, on average during a year). Generally, as reviewed by Haverty (1977) on 112 termite species, the proportion of soldiers in a colony is low in all the investigated species.

In social hymenopterans, the low proportion of reproductive castes was often discussed in terms of conflicts among colony members (Bourke and Franks, 1961). Conflict among reproductives is also suggested to exist in termite sociality since the artificial addition of extra reproductives resulted in cannibalism among reproductives (Lüscher, 1961). In contrast, the adaptive meaning of low soldier proportion has been discussed based on the results of experimental manipulations. Basically, a colony needs soldiers because soldier is the only caste that can intercept attacks by enemies (mainly predators) (Noirot and Darlington, 2000). However, the possession of soldiers in colonies should incur some costs, since soldiers cannot feed by themselves but require help from workers (Noirot and Darlington, 2000; Korb and Hartfelder, 2008). Actually, an excess of soldiers caused high mortality of nestmates in *Coptotermes formosanus* (Haverty, 1979). Thus, maintaining the appropriate proportion of soldiers is necessary for termite colonies. However, only a few studies on the adaptive significance of caste proportion have been reported in termites.

The most famous discoveries on the regulatory mechanisms of reproductive caste differentiation in termites were a series of studies on the differentiation of secondary reproductives of *Kalotermes flavicollis* performed by Lüscher (1952, 1961) in the middle of 20th century. He clarified the developmental pathway of the focal species and the fact that workers had the potential to differentiate into both secondary reproductives and soldiers. Moreover, his subsequent studies revealed that workers differentiated into secondary reproductives or soldiers after the removal of primary reproductives or soldiers, respectively, from the colony (Lüscher, 1960, 1961, 1974). These results suggested that the caste differentiation was repressively regulated by other colony members (castes) probably through pheromones. Furthermore, the differentiation of secondary reproductives was not inhibited when the anus of primary reproductives was sealed, indicating that some substances were transferred through proctodeal trophallaxis (Lüscher, 1955, 1974). In *K. flavicollis*, fewer alates were differentiated when nymphs were isolated with the royal pair than in groups of orphan nymphs (Springhetti, 1969, 1971, 1972). This inhibitory effect occurred only when the king and queen had directly contacted with nymphs, suggesting that the direct communications such as stomodeal and/or proctodeal trophallaxis, or grooming would be responsible for it.

**Figure 2:**
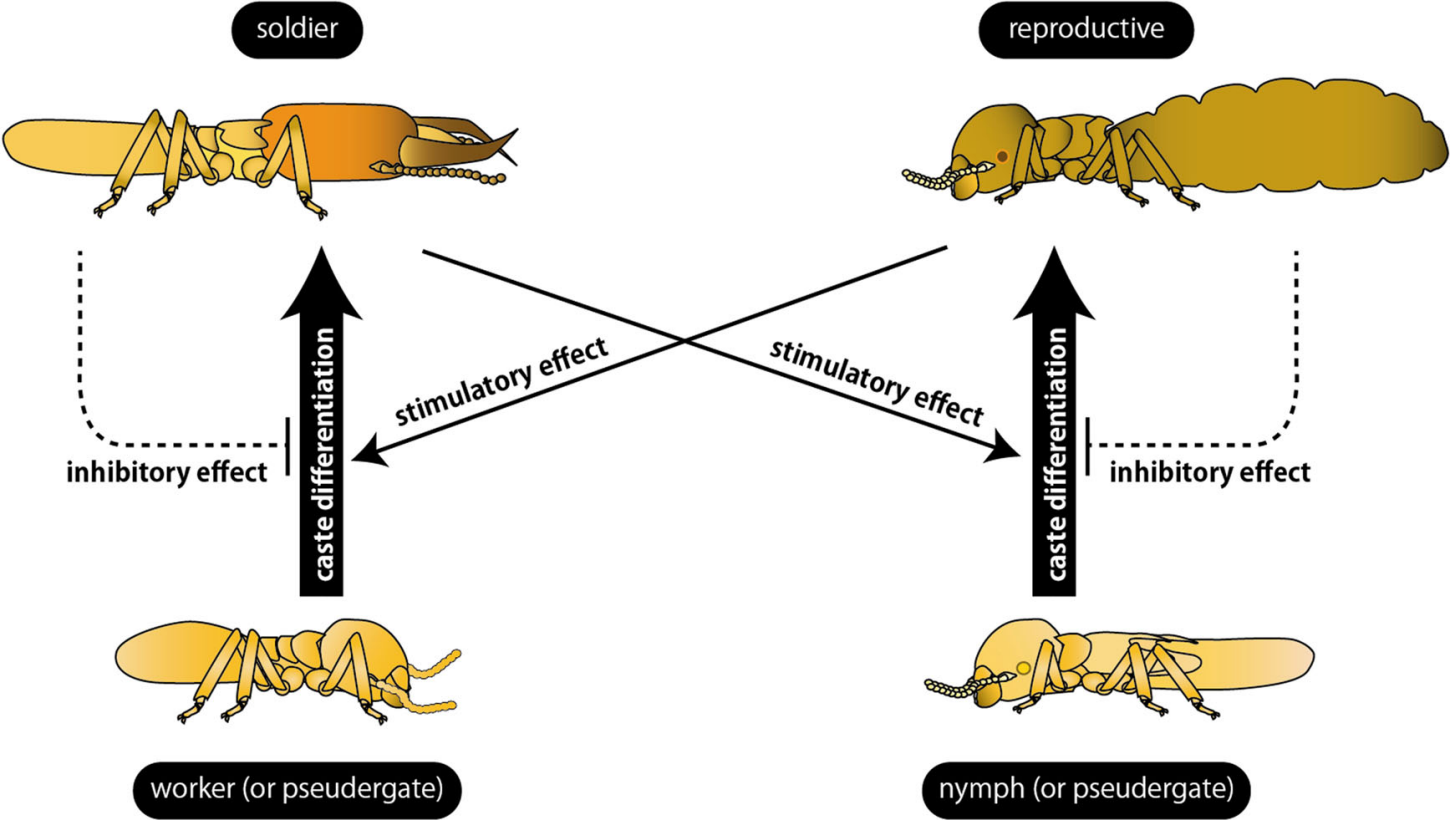
**Summary of the regulation of caste differentiation by social interactions.** Differentiation into reproductives and soldiers (bold arrows) are stimulated by different castes (thin arrows) and inhibited by the same castes (broken arrows). Note that the effect of secondary reproductives on soldier differentiation is still an open question (see text for details).

In contrast to the inhibitory effects of primary reproductives, the alate production was shown to be accelerated by the presence of soldiers in *Zootermopsis nevadensis* (Lüscher, 1975). Similarly, the differentiation of secondary reproductives of *K. flavicollis* was accelerated by soldiers (Springhetti, 1969). Both cases indicate that the differentiation to reproductives seemed to be induced by the presence of soldiers (Figure 2).

The inhibition of soldier differentiation by soldiers was first reported in incipient colonies of *Z. angusticollis*. Most of the incipient colonies possessed only one soldier for a long time. Castle (1934) discovered that, when a soldier was introduced into another incipient colony before the first soldier was differentiated, soldier differentiation was suppressed. When the soldier was removed from an incipient colony, another workers started to differentiate into a new soldiers. By using individuals collected from mature colonies, the inhibitory effects of soldiers on the additional soldier differentiation were demonstrated in *K. flavicollis* (Figure 2) (Springhetti, 1969, 1985). Lefeuve and Bordereau (1984) firstly reported the chemical characteristics of soldier pheromone using *Nasutitermes lujae*. They revealed that not only living soldiers but also chemical components extracted from soldiers using organic solvent inhibited the soldier differentiation. These inhibitory effects did not occur unless workers directly contacted with the soldier extracts, suggesting that the pheromone suppressing additional soldier differentiation was a non-volatile, non-polar substance. Furthermore, Okot-Kotber et al. (1991) reported that soldier extracts of *Reticulitermes flavipes* inhibited soldier differentiation in termite species belonging to a different family, suggesting that the inhibitory mechanisms are shared among termite lineages.

On the other hand, in *N. lujae* and *Cubitermes fungifaber*, the soldier differentiation occurred more frequently in the groups of workers reared with primary reproductives than those without reproductives (Bordereau and Han, 1986). Similar effects of reproductives on the additional soldier differentiation were found in *K. flavicollis* (Springhetti, 1969, 1970) and *Z. nevadensis* (Maekawa et al., 2012). Generally in termites, the soldier differentiation is stimulated by the presence of primary reproductives and suppressed by other soldiers (Figure 2). The presence of secondary reproductives is thought to stimulate the soldier differentiation as well as primary reproductives, although the direct evidences have not been reported. In general, the caste differentiation appears to be inhibited by the same caste while induced by a different caste, probably through pheromone actions (Bordereau, 1985; Noirot, 1991).

## Primer pheromones mediating social interactions

The term “pheromone” was firstly defined by Karlson and Lüscher (1959), who suggested that, in termites, some substances inhibit the additional differentiation of secondary reproductives, which was repressed by interactions between primary reproductives and workers. Since then, the pheromones controlling caste differentiation in termites had not been identified. However, recent techniques of chemical analyses applied to ecological phenomenon have contributed to the identification of caste-regulating pheromones in termites. For example, n-butyl-n-butyrate and 2-methyl-1-butanol were reported as the essential components of queen pheromones in *R. speratus* (Matsuura et al., 2010).

For the soldier differentiation, two related terpenoids were identified as dominant components from the soldier head extracts in *R. flavipes*: γ-cadinene and its aldehyde, γ-cadinenal (Tarver et al., 2009). The subsequent analyses revealed that γ-cadinenal worked as a pheromone inhibiting the soldier differentiation (so-called “soldier pheromone”) and γ-cadinene as a pheromone promoting the soldier differentiation (Tarver et al., 2011). However, these candidate pheromone substances were not detected from some populations of the focal species, suggesting that the synthesis of these substances depends on populations and/or seasons (Perdereau et al., 2010). In addition to these, β-selinene was identified as a major compound in the soldier head extracts in *R. speratus* acting as an aggregation pheromone for colony members (Nguyen et al., 2011). They suggested that this substance might be involved in the inhibition of soldier differentiation from workers because the interactions among colony members might be accelerated by aggregation. Actually, the direct contact of soldiers with workers inhibits the additional soldier differentiation from workers in *Coptotermes formosanus* (Park and Raina, 2003; Dong et al., 2009).

Recent advances in chemical analyses have eventually enabled us to identify queen and soldier pheromones in termites that were predicted by Karlson and Lüscher (1959). These advances will accelerate researches on the regulatory mechanisms of caste ratio. In future studies, many other functional compounds will be identified, so that it will be possible to examine the regulatory mechanisms by those pheromone chemicals, including the downstream reception and endocrinological pathways, are universal across termite taxa.

## Juvenile hormone—an endocrine mediator between the social interaction and the soldier-caste differentiation

### JH-titer quantification applied to termite biology

**Figure 3:**
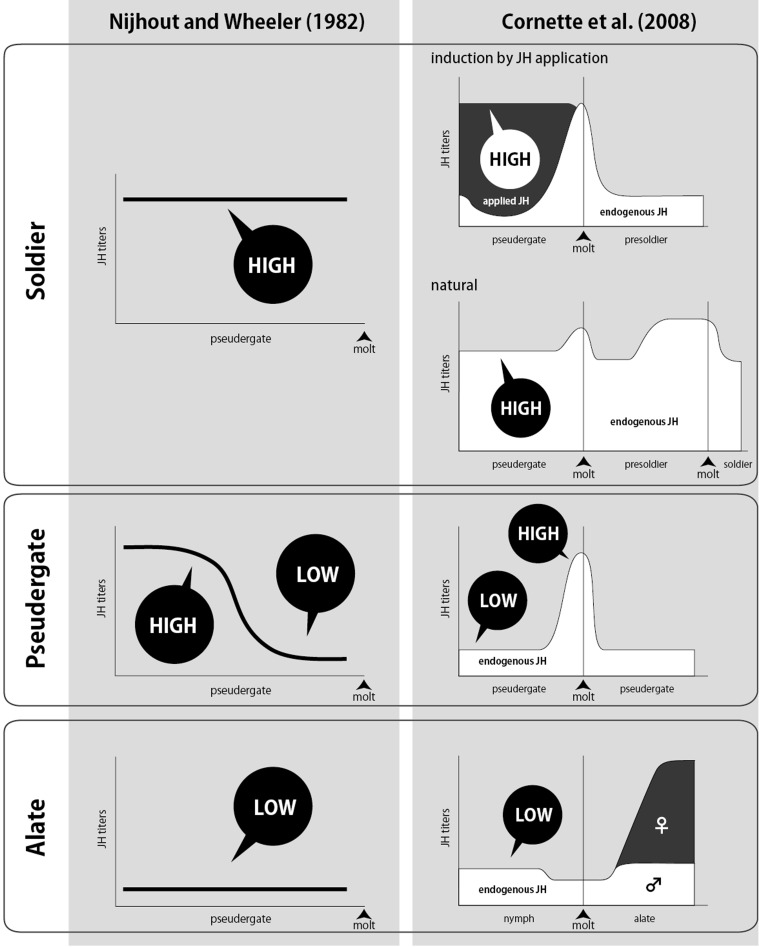
**Schematic drawings of classical (Nijhout and Wheeler, 1982; left) and recent (Cornette et al., 2008; right) models on the transitions of JH titer (vertical axis) during intermolt period of pseudergate (horizontal axis) before caste differentiation.** Caste names at the far left indicate the future castes determined by the JH patterns. According to Cornette et al. (2008), changes in JH titers during the pseudergate stage before the differentiation into soldiers or alates were consistent with classical models. There were some differences between the model and measured titers in the case of stationary molts.

Early studies demonstrated that the transplantation of corpora allata (CA; juvenile hormone-producing endocrine glands) induced caste differentiation of recipient workers in *K. flavicollis* (Lüscher, 1952, 1960). CA from soldiers, reproductives and late-stage nymphs induced the soldier differentiation, whereas CA from workers and early-stage nymphs induced the differentiation into reproductives. These results suggest that juvenile hormone (JH) plays important roles in the caste differentiation, and the JH synthesis activity in CA differs among castes. In addition to the CA transplantation, the applications of ectopic JH analogs (JHAs) to workers also induced the soldier differentiation in many termite species, indicating that an increase in JH titer stimulated differentiation into soldiers (Howard and Haverty, 1979). Thus, JH has been recognized as the most important endocrine factor for caste differentiation, and a model of caste differentiation by JH action was proposed, in which high JH titer induces soldier differentiation whereas low JH titer causes differentiation into alates (Nijhout and Wheeler, 1982; Figure 3).

Although classical studies suggested that the patterns of JH-titer transitions during intermolt determined the future caste differentiation, precise measurements of JH titer were not performed until the establishment of JH quantification methods (Zera, 2007). So far, three major methods for JH quantification have been reported: radioimmunoassay (Greenberg and Tobe, 1985; Goodman et al., 1990), gas chromatography-mass spectrometry (GC-MS) (Shu et al., 1997), liquid chromatography-mass spectrometry (LC-MS) (Westerlund and Hoffmann, 2004). The JH quantification by GC-MS was developed earlier than those using LC-MS. For the JH quantification to be a routine lab procedure, however, GC-MS analysis has some disadvantages due to the requirement of many samples and mass consumption of harmful organic solvents for sample preparation (Bergot et al., 1981; Rembold and Lackner, 1985). Recently, a rapid and accurate method for JH quantification by LC-MS was developed to quantify insect JHs (e.g., JHI, II, and III) and their metabolites (Westerlund and Hoffmann, 2004). By applying this method, Cornette et al. (2008) and Gotoh et al. (2008) established the quantification of JH titers in a termite *Hodotermopsis sjostedti*. Cornette et al. (2008) further analyzed the patterns of JH titers during differentiation into reproductives and soldiers in *H. sjostedti*, indicating that JH titers of pseudergates prior to soldier differentiation were constantly high, whereas, those of nymphs before molts into alates remained low (Figure 3). These two JH transitions leading to the soldier and alate differentiations are consistent with the patterns proposed by Nijhout and Wheeler (1982), although there are minor differences in the patterns leading to the stationary molt (repeating the same instar) to pseudergates (Figure 3). It was also demonstrated that JH titers of nymphs before alate differentiation were low in *Cryptotermes secundus* (Korb et al., 2009a) and *Reticulitermes speratus* (Maekawa et al., 2010).

As for the regulations of the hemolymphatic JH titer in insects, it is known that JH titer is not only regulated by the activity of JH synthesis in CA and secretion from CA, but also by other biological processes such as sequestration and degradation (Gilbert et al., 2000; Nation, 2008). Actually, the transcription levels of *hexamerins* (a factor related to JH sequestration) were shown to be up-regulated during soldier differentiation (Scharf et al., 2005, 2007; Cornette et al., 2013), so that the sequestration process is suggested to modify JH titer during the caste differentiation.

### Soldier presence affects the worker JH titer

Based on the fact that JH is the most important factor involved in the caste differentiation in termites, it was considered that the JH actions mediate the effects of interactions among colony members on the caste differentiation (Henderson, 1998). By applying the technique of GC-MS analysis in *Coptotermes formosanus*, it was shown that the isolation of workers from soldiers increased the worker JH titers, suggesting that the presence of soldiers may repress the JH titer of nestmates (Mao et al., 2005; Park and Raina, 2005). Since these studies did not employ the application of JH or JH analogs, the observed effects were suggested to occur under natural conditions.

**Figure 4:**
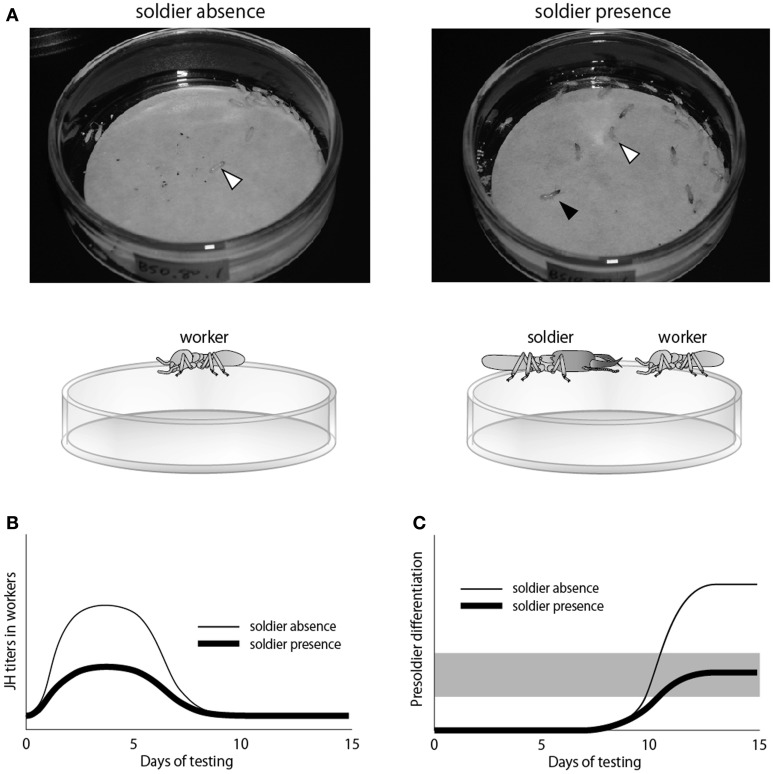
**A study examining the relationship between social interactions and JH titers in termites (Watanabe et al., 2011). (A)** Experimental designs to obtain workers (white arrowheads) under the absence/presence of soldiers (black arrowhead) put on petri dishes with filter papers containing JHIII. The JH titers of workers reared by this method were quantified along the time course. **(B)** JH-titer transitions in workers during the soldier induction experiment. **(C)** Induction rates of presoldier differentiation under the absence/presence of soldiers. The gray shading indicates the range of soldier ratio observed in nature.

The JH quantification method by LC-MS that was performed in *H. sjostedti* (Cornette et al., 2003) was also applied to analyze precise effects of the soldier presence on worker JH titers during the soldier differentiation in *R. speratus* (Watanabe et al., 2011; Figure 4). The results of this study in which JH titers of JH-treated workers reared with/without soldiers were quantified (Figure 4A), revealed that, under the absence of soldiers, the JH application rapidly increased the worker JH titers (Figure 4B), resulting in the elevation of presoldier differentiation ratio from workers (Figure 4C). On the other hand, under the presence of soldiers, the rapid increase in worker JH titers by JH application was suppressed (Figure 4B), leading to the low induction rates (Figure 4C). These results suggested that the soldier differentiation triggered by the JH application was suppressed by the soldier presence, probably by lowering the worker JH titer. This suppression by the soldier presence occurred very rapidly (within a few days), suggesting the rapid and accurate regulation of soldier-caste ratio in termites.

### Soldier-specific morphogenesis affected by social interactions

Extremely elongated mandibles used to bite or flick enemies (mechanical defense), or frontal glands discharging aversive and/or toxic substances (chemical defense) toward enemies (Deligne et al., 1981; Prestwich, 1984) were formed through the distinctive morphogenetic events during the process of soldier differentiation (Noirot, 1969; Weesner, 1969). This process can be induced artificially by using JH or JHA, which initiates the soldier differentiation (Miura et al., 2003; Šobotník et al., 2010).

**Figure 5:**
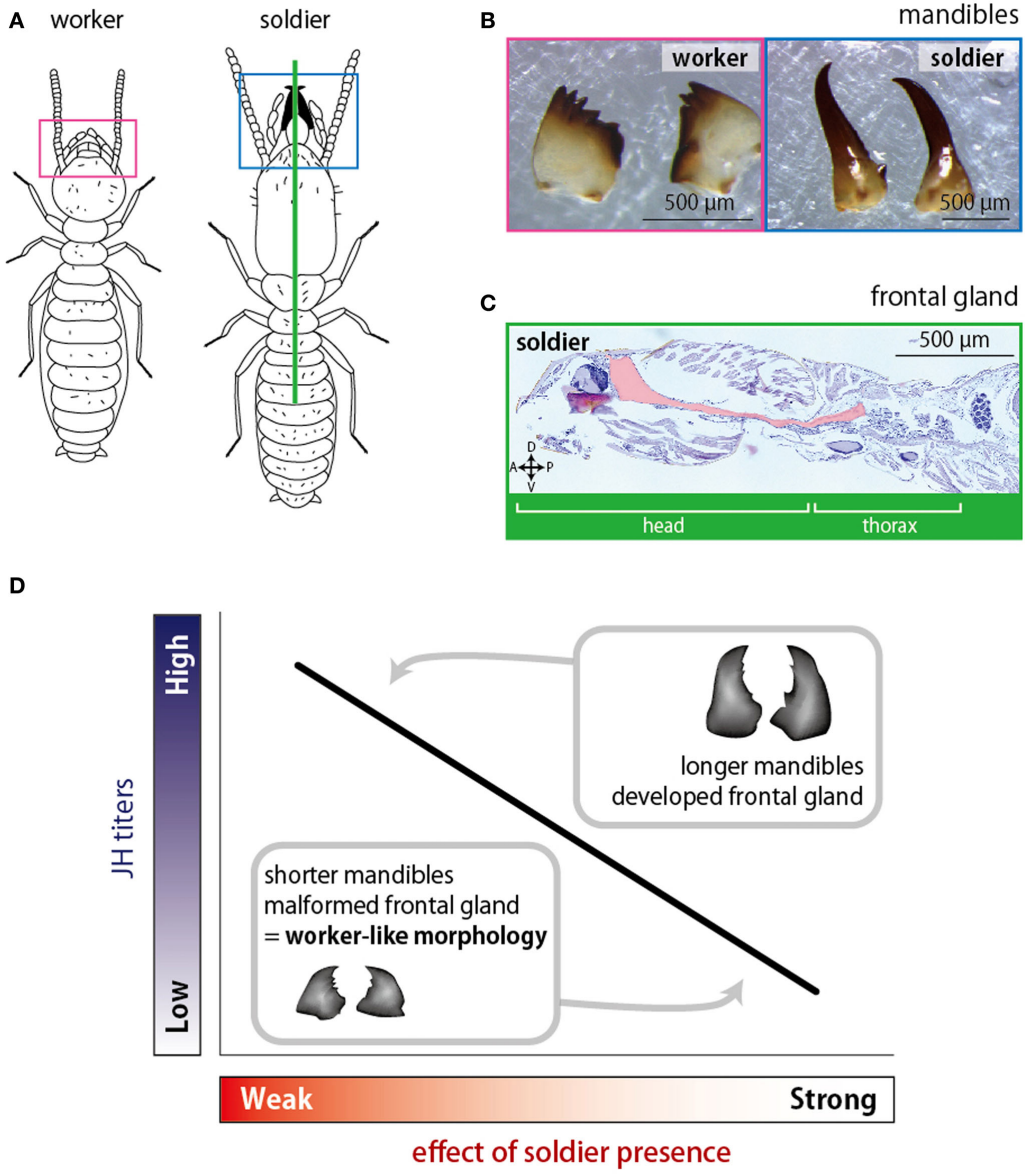
**The soldier effects on morphogenesis shown by Tsuchiya et al. (2008) and Watanabe and Maekawa (2012). (A)** Illustrations of a soldier and a worker of *Reticulitermes speratus*. **(B)** Mandibles of a worker (left) and a soldier (right). **(C)** A Median section of a soldier. The frontal gland invaginates into the head and thorax (indicated by pink). **(D)** Relationships between the effects of soldier presence and the JH titers of workers. When the soldier effect is weak, the JH titer rises resulting in the elongation of mandibles. Under the strong soldier effect, low JH titer repress the mandibular elongation.

In order to examine the relationship between JH titer and mandibular length, various concentrations of JH were applied to workers in *Reticulitermes speratus*, in which soldiers possess elongated mandibles (Tsuchiya et al., 2008; Figures 5A,B). The results showed that mandibular lengths of induced presoldiers depended on the concentrations of applied JH. Namely, higher concentrations of JH induced presoldiers with longer mandibles while lower concentrations induced presoldiers with shorter mandibles. Interestingly, the resultant mandibular lengths were also affected by the presence of soldiers even under the same JH concentration; workers coexisted with soldiers molted to presoldiers with shorter mandibles while those without soldiers molted to presoldiers with longer mandibles. These results strongly suggest that the presence of soldiers affects the developmental processes in the soldier differentiation by controlling JH titer (Tsuchiya et al., 2008). Actually, a subsequent work quantifying the endogenous JH titer in the same species supported this idea (Watanabe et al., 2011).

In some species of rhinotermitids, the frontal gland seen in soldiers (Figure 5C) is also formed during the soldier differentiation induced by JH applications (Lelis and Everaerts, 1993; Watanabe and Maekawa, 2008; Šobotník et al., 2010). In *R. speratus*, Watanabe and Maekawa (2012) showed that the degree of frontal-gland formation depended on the concentrations of applied JH; higher JH concentration induced well-developed glands whereas lower one did not. It was also suggested that the soldier presence suppressed the frontal-gland formation via the regulation of JH titer (Figure 5D). These studies focusing on the development of soldier characters indicated that interactions among individuals did not only affect the caste differentiation but also the caste-specific morphogenesis. In these cases, JH titers in workers might be suppressed to lower levels by the presence of soldiers (Figure 5D). Since the social interactions are suggested to affect the expression patterns of genes that were shown to regulate the soldier-specific morphogenesis (Koshikawa et al., 2002, 2003, 2005, 2010; Hattori et al., 2013; Toga et al., 2012, 2013), the relationships between those gene expression patterns and social interactions will be clarified sooner or later.

## Molecular bases underlying the social interactions

During the last decade, many pioneering works in termites related to the new study area “sociogenomics” have been reported (Robinson, 1999; Robinson et al., 2005). By large-scale gene expression analyses (such as differential display, macro-array, cDNA subtraction and representation difference analysis) followed by functional assays such as RNA interference (RNAi), the proximate mechanisms of caste differentiation have been extensively analyzed, leading to the identifications of genes involved in the termite sociality (Miura and Scharf, 2011). These can be the candidate factors involved in the social interactions affecting the caste differentiation.

The caste-specific gene that was firstly identified is *Soldier-specific protein 1* (*SOL1*) in *Hodotermopsis sjostedti*, that was expressed exclusively in soldiers (Miura et al., 1999). Although the function of SOL1 remains unknown, the encoded protein belongs to the lipocalin protein family (Miura, 2004, 2005; Flower, 2000). Considering the general functions of lipocalins in signal transductions, there is a possibility that SOL1 might be involved in communications among colony members such as a carrier of soldier pheromone (Miura and Scharf, 2011).

Among genes involved in JH-related pathway, *hexamerins* and *cytochrome P450s* are well studied (Miura and Scharf, 2011). Hexamerins are one of the groups of JH-binding proteins (Gilbert et al., 2000), and *hexamerin* (*Hex*) genes were expressed prominently in fat bodies of workers during the soldier differentiation in *Reticulitermes flavipes* (Scharf et al., 2005; Zhou et al., 2006a). The *Hex*-knockdown by RNAi induced the soldier differentiation, suggesting that *Hex* genes play an important role in the soldier differentiation, by regulating JH titer in workers (Zhou et al., 2006b). Also in *Hodotermopsis sjostedti*, the *Hex* expressions were up-regulated in the course of soldier differentiation (Cornette et al., 2013), suggesting that hexamerin-based regulatory mechanisms of JH are widespread in termites. Cytochrome P450s are heme-containing oxidative enzymes which catalyze the syntheses and metabolisms of JHs as well as those of ecdysteroids and some pheromones in insects (Feyereisen, 1999; Scott and Wen, 2001). The expression levels of *cytochrome P450* genes significantly up-regulated during the soldier differentiation in *H. sjostedti* (Cornette et al., 2006) and *R. flavipes* (Zhou et al., 2006c). Therefore, hexamerins and P450s can be candidate mediators between social interactions and physiological influences, as a study suggested that hexamerins played a role in the JH-titer regulation under the influences of nutritional and temperature conditions (Scharf et al., 2007).

Recently, a gene, called “*Neofem2*” that affects the mode of social interaction between a queen and workers was discovered in *Cryptotermes secundus* (Korb et al., 2009b). Under the presence of a queen, workers did not perform head-butting behavior nor differentiate into secondary reproductives. However, when the gene *Neofem2* was silenced in the primary queen, workers performed the head-butting behavior and started to reproduce. This result indicated that *Neofem2* mediated the reproductive division of labor between queen and workers. *Neofem2* encodes β-glycosidase, which was reported as a pheromone involved in egg recognition and communication in termites and cockroaches (Cornette et al., 2003; Matsuura et al., 2009). However, it should be noted that the mechanisms connecting the behavioral regulations and the reproductive differentiation remains to be understood.

## Conclusion and perspectives

In brief, our primary purpose of this review is to summarize the mechanisms of social interactions affecting the regulations of caste differentiation, especially by focusing on the physiological regulations in soldier differentiation. In this article, many studies across various biological processes from the pheromone communications among castes to the alterations of postembryonic development are overviewed, although there are still “missing links” between those processes we here reviewed. For example, the mechanism connecting between social interactions and physiological changes still remains understood. The social physiological mechanisms known in hymenopterans such as the usage of odorant binding proteins (Gp-9) in the fire ant (Gotzek and Ross, 2007) will provide us some hints to complement the missing links. Actually examples of the similar mechanisms related to the social physiology are shared between hymenopterans and termites. In honeybee, vitellogenins are involved in the transition from nursing to foraging in workers (Nelson et al., 2007), while in termites, hexamerins regulate the soldier differentiation (Zhou et al., 2006b; Cornette et al., 2013). Although these two groups of proteins belong to the different families, both are known to be involved in the control of JH titer in insects (Gilbert et al., 2000; Nation, 2008). Therefore, these studies suggest that the sequestration process of JH may be important for the phenotypic transitions such as caste differentiation and age polyethism in social insects. Thus, the in-depth understanding on the complex social interactions in termites will contribute to the deeper understanding of principles in social physiology.

Recently, innovations in a new interdisciplinary science, sociogenomics, and chemical ecology are shedding light on the proximate mechanisms of social interactions regulating caste differentiation, leading to the identifications of candidate factors involved in these mechanisms such as primer pheromones, carrier proteins of pheromones and factors regulating JH titers. Recent advances in genomics, transcriptomics, and bioinformatics are also pushing forward the understanding social physiology. For example, the first termite genome sequencing project has been done in *Zootermopsis nevadensis* (http://termitegenome.org/). Furthermore, genomics and transciptomics in other termite species are proposed by the two major projects in arthpods: the i5k project (http://arthropodgenomes.org/wiki/i5K; including 25 termite species) and 1KITE project (http://www.1kite.org/index.html; including 4 termite species). The future contributions of these projects, together with the achievements in previous studies (e.g., The Honeybee Genome Sequencing Consortium, 2006; Bonasio et al., 2010), will lead to the comprehensive understandings of insect sociality. For example, comparative genomics may reveal the commonalties and diversities of social physiology seen in various lineages of social insects.

### Conflict of interest statement

The authors declare that the research was conducted in the absence of any commercial or financial relationships that could be construed as a potential conflict of interest.
